# Neuroprotective effects of intravenous immunoglobulin are mediated through inhibition of complement activation and apoptosis in a rat model of sepsis

**DOI:** 10.1186/s40635-016-0114-1

**Published:** 2017-01-05

**Authors:** Figen Esen, Gunseli Orhun, Perihan Ergin Ozcan, Evren Senturk, Melike Kucukerden, Murat Giris, Ugur Akcan, Canan Ugur Yilmaz, Nurcan Orhan, Nadir Arican, Mehmet Kaya, Sema Bilgic Gazioglu, Erdem Tuzun

**Affiliations:** 1 Department of Anesthesiology, Istanbul Faculty of Medicine, Istanbul University, Capa-Fatih, 34039 Istanbul, Turkey; 2 Neuroscience, Institute of Experimental Medicine, Istanbul University, Istanbul, Turkey; 3 Department of Physiology, Istanbul Faculty of Medicine, Istanbul University, Istanbul, Turkey; 4 Department of Forensic Medicine, Istanbul Faculty of Medicine, Istanbul University, Istanbul, Turkey; 5 Immunology, Institute of Experimental Medicine, Istanbul University, Istanbul, Turkey

**Keywords:** Sepsis, Immunoglobulins, Cecal ligation and puncture, Complement activation, Apoptosis

## Abstract

**Background:**

Intravenous (IV) immunoglobulin (Ig) treatment is known to alleviate behavioral deficits and increase survival in the experimentally induced model of sepsis. To delineate the mechanisms by which IVIg treatment prevents neuronal dysfunction, an array of immunological and apoptosis markers was investigated.

**Methods:**

Sepsis was induced by cecal ligation perforation (CLP) in rats. The animals were divided into five groups: sham, control, CLP + saline, CLP + immunoglobulin G (IgG) (250 mg/kg, iv), and CLP + immunoglobulins enriched with immunoglobulin M (IgGAM) (250 mg/kg, iv). Blood and brain samples were taken in two sets of experiments to see the early (24 h) and late (10 days) effects of treatment. Total complement activity, complement 3 (C3), and soluble complement C5b-9 levels were measured in the sera of rats using ELISA-based methods. Cerebral complement, complement receptor, NF-κB, Bax, and Bcl-2 expressions were analyzed by western blot and/or RT-PCR methods. Immune cell infiltration and gliosis were examined by immunohistochemistry using CD3, CD4, CD8, CD11b, CD19, and glial fibrillary acidic protein antibodies. Apoptotic neuronal death was investigated by TUNEL staining.

**Results:**

IVIgG and IgGAM administration significantly reduced systemic complement activity and cerebral C5a and C5a receptor expression. Likewise, both treatment methods reduced proapoptotic NF-κB and Bax expressions in the brain. IVIgG and IgGAM treatment induced considerable amelioration in glial cell proliferation and neuronal apoptosis which were increased in non-treated septic rats.

**Conclusions:**

We suggest that IVIgG and IgGAM administration ameliorates neuronal dysfunction and behavioral deficits by reducing apoptotic cell death and glial cell proliferation. In both treatment methods, these beneficial effects might be mediated through reduction of anaphylatoxic C5a activity and subsequent inhibition of inflammation and apoptosis pathways.

**Electronic supplementary material:**

The online version of this article (doi:10.1186/s40635-016-0114-1) contains supplementary material, which is available to authorized users.

## Background

Septic encephalopathy is characterized with delirium, coma, and seizure and is a cause of cognitive dysfunction, morbidity, and mortality in critical illness [1]. Longer mechanical ventilation, ICU stay, and post-ICU cognitive dysfunction were reported in sepsis survivors who demonstrated delirium as a major sign of septic encephalopathy [2]. There are no specific diagnostic tests and treatment methods for brain dysfunction, and the exact mechanism of brain involvement in sepsis is not clear. However, endothelial dysfunction and blood–brain barrier (BBB) failure were found responsible as a mechanism of injury [3]. Treatment modalities targeting inflammation have been suggested for the prevention of septic encephalopathy. Among inflammation factors, the complement system is a particularly well-known participant in sepsis and anti-C5a antibody treatment has been shown to attenuate the BBB failure in septic animals [4, 5]. In our previous study, intravenous immunoglobulin (IVIg) improved the integrity of the BBB and inhibited cecal ligation and perforation (CLP)-induced symptoms of sickness behavior in rats [6]. In this experimental trial, we aimed to delineate mechanisms by which IVIg treatment prevents neuronal dysfunction. The complement system is activated by both antibodies (classical pathway) and microorganisms (lectin and alternative pathways) leading to the formation of membrane attack complexes (C5b-9), which lyse and destroy target cells. During the activation of complement pathways, anaphylatoxins C3a and C5a are released and interact with their receptors leading to the activation of inflammation, apoptosis, and gliosis pathways [4]. Given the well-known significance of the complement system in sepsis and the well-established regulatory effect of IVIg on complement activation, we hypothesized that IVIg treatment improves septic encephalopathy through the inhibition of complement-mediated neuronal destruction. To test this hypothesis and find out the specific complement factors involved in septic encephalopathy, we measured the expression levels of an array of complement factors (C1q, C3, and C9 for evaluation of classical and common complement pathways; C3a, C5a, and their receptors for anaphylatoxic component of the complement system) and evaluated expression alterations in parallel to cerebral apoptosis and gliosis in the brain samples of septic rats.

## Methods

### Animals

We used male Sprague–Dawley rats (200–250 g). The animals stayed in groups under normal conditions with access to enough food and water ad libitum. The procedures of the study were approved by the Local Ethics Committee for Animal Experimentation (101/2013 September).

Experimental groups were assigned as five groups: control (*n* = 16), sham (*n* = 16), CLP + saline (*n* = 16), CLP + immunoglobulin G (IgG) (250 mg/kg, iv) (*n* = 16), and CLP + immunoglobulin enriched with immunoglobulin M (IgGAM) (250 mg/kg, iv) (*n* = 16). Blood and brain samples were taken in two sets of experiments after CLP to see the early (24 h) and late (10 days) effects of treatment.

### CLP procedure

To assess cerebral complement expression alterations, apoptosis, and gliosis induced by sepsis and to delineate the impact of IVIg treatment on these parameters, a CLP-based sepsis model was performed as described previously [7]. After intraperitoneal ketamine administration (100 mg/kg), a longitudinal midline abdominal incision was made with a scalpel. A small scissor was used to extend the incision and gain entry into the peritoneal cavity. The cecum was isolated and exteriorized with blunt anatomical forceps; care was taken not to breach or damage the mesenteric blood vessels. To induce high-grade sepsis, the cecum was ligated and perforated by a single through-and-through puncture midway between the ligation using an 18-G needle. After removing the needle, a small amount of feces was extruded from both the mesenteric and antimesenteric penetration holes and the cecum was relocated into the abdominal cavity. Following the closure of the wound, the animals were resuscitated by injecting warmed saline (37 °C; 5 ml per 100 g body weight) subcutaneously. The sham-operated rats underwent the same procedure, except for the ligation and perforation of the cecum. The control rats received neither any surgical intervention nor IVIg treatment. At the early stage (24 h) of the sepsis model, the survival rate of the CLP + saline group was 6/8 (75%), whereas in the control, sham, CLP + IgG, and CLP + IgGAM group, the survival rates were 8/8 (100%). In the late stage (10 days), the survival rates were 5/8 (62.5%) in the CLP + saline group, 7/8 (87.5%) in the CLP + IgG and CLP + IgGAM groups, and 8/8 (100%) in the control and sham groups.

### Administration of immunoglobulins

The animals were given human IgG, 250 mg/kg (Octapharma; Vienna, Austria), or IgGAM, 250 mg/kg (Pentaglobin; Biotest, Dreieich, Germany), intravenously via penile vein 5 min after the CLP procedure. After the IV injection, the animals were placed back in their cages for recovery.

### ELISA for serum total complement activity and complement levels

To assess the effects of IVIg treatment on systemic complement activity and to validate the complement activity inhibiting effect of IVIg, serum total complement activity (CH50), C3, and soluble C5b-9 levels were measured by ELISA kits (Neoscientific, Cambridge, MA, USA), as per the manufacturer’s instructions. Optical density was measured at 450 nm, and concentrations were calculated by referring to a standard curve.

### Real-time PCR

Expression levels of major complement factors, complement regulators, and apoptosis factors in septic encephalopathy model were evaluated by real-time PCR. Total RNA from each brain sample was isolated using TRIzol Reagent (Invitrogen, Carlsbad, CA, USA), and RNA was quantified by OD 260. Four micrograms of total messenger RNA (mRNA) were reverse transcribed using superscript II reverse transcriptase and oligo dT primer (Invitrogen). Specific primers (Additional file 1: Table S1) were optimized using primer3 software and generated by Qiagen (Hilden, Germany). The specificity of the primers was verified with a Blast search through NCBI. The quantitative real-time PCR reactions were performed with the SYBR Green kit (Roche Diagnostics, Mannheim, Germany) using 2 μl of cDNA and 0.6 μl of each primer in a 20-μl final volume. Quantitative PCR was performed using Light Cycler (Roche Diagnostics) for 40 cycles at 95 °C for 15 s and at annealing temperatures of 60 °C for 20 s and 72 °C for 30 s. All samples were studied as duplicates, and three housekeeping genes were used as reference genes. Data were analyzed according to ΔΔCt method, and the results were expressed as relative mRNA levels.

### Immunoblotting analyses

To confirm the real-time PCR results of most crucially altered complement and apoptosis factors at the protein level, immunoblotting experiments were conducted. Twenty micrograms of each brain lysate was loaded and separated by 4–20% SDS–polyacrylamide gradient gel electrophoresis and then transferred to 0.45-μm polyvinylidene fluoride membranes (100 V, 80 min). After blocking for 1 h in Phosphate buffered saline with tween 20 (PBST) (10 mM sodium phosphate, 0.9% NaCl, and 0.1% Tween 20) containing 5% non-fat dry milk, blots were incubated overnight at 4 °C with the primary antibodies (Additional file 2: Table S2) in PBST containing 3% non-fat milk. The blots were washed four times with PBST (40 min) and incubated for 1 h with horseradish peroxidase-conjugated secondary antibody (Santa Cruz Biotechnology, Santa Cruz, CA, USA) in PBST containing 3% non-fat dry milk. Immunoreactivity of the protein bands were detected by enhanced chemiluminescent autoradiography (ECL kit, Amersham Pharmacia Biotech, Piscataway, NJ). A molecular weight standard (Bio-Rad Laboratories, Hercules, CA) was loaded in the last lane of each gel to assess relative molecular mass of detected bands. The immune blot bands were quantified through measurement of band intensity with ImageJ software using the same pixel scale for all pictures. Band intensities were normalized by β-actin expression and expressed as arbitrary units.

### TUNEL and immunohistochemistry

The presence and intensity of infiltrating immune cells, apoptosis, and gliosis were evaluated with immunohistochemical methods. Brain samples were first evaluated by standard hematoxylin and eosin (H&E) staining. To investigate the presence of potential infiltrating immune cells and reactive gliosis, immunohistochemistry studies were performed. Brain samples were first treated with 4% paraformaldehyde overnight at 4 °C, immersed in 40% sucrose overnight at 4 °C, and subsequently snap frozen in liquid nitrogen. Seven-micrometer-thick frozen sections were serially incubated with 0.3% H_2_O_2_ for 20 min, 10% goat serum for 1 h, and primary antibodies (Additional file 2: Table S2) overnight at 4 °C. The sections were then incubated in biotinylated goat anti-human IgG (1:2000, Vector Laboratories, Burlingame, CA), and the immunoreactivity was developed by serial incubation with avidin–biotin peroxidase (Vector Laboratories) for 1 h and diaminobenzidine [8]. TUNEL staining was done with an apoptosis detection kit (Merck Millipore, Darmstadt, Germany) according to the manufacturer’s instructions. Rat spleen sections were used as a positive control in all experiments. The presence of immune cells, apoptosis, and gliosis were visualized by two independent blinded observers.

#### Statistics

Serum complement factor and activity levels, brain mRNA expression levels measured by real-time PCR, and normalized brain protein expression levels measured by immunoblot experiments were compared among different treatment arms by using ANOVA followed with Tukey adjustment for multiple pairwise comparisons. *p* < 0.05 was considered statistically significant.

## Results

### Reduced C5a activity in IVIgG- and IgGAM-administered rats

One day after treatment, the IVIgG- and IgGAM-administered rats showed significantly reduced CH50 levels and significantly increased serum C3 and soluble C5b-9 levels. By contrast, on day 10, CH50, C3, and C5b-9 levels were comparable among groups (Additional file 3: Figure S1).

**Fig. 1:**
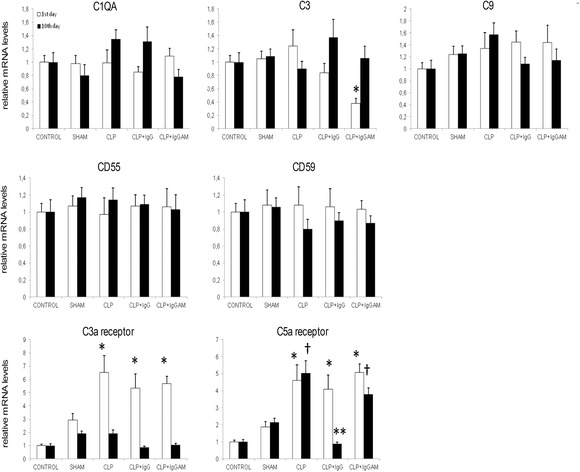
Brain mRNA expression levels of complement factors, complement inhibitors, and complement receptors evaluated by real-time PCR and expressed as fold changes relative to the control group. *^†^ *p* < 0.05; **p < 0.01. In the C3 panel, * denotes significant differences between the CLP + IgGAM group vs the other groups; in the C3a receptor and C5a receptor panels, * denotes significant differences between the CLP, CLP + IgG, and CLP + IgGAM groups vs the control and sham groups; and in the C5a receptor panel, † denotes significant differences between CLP and CLP-IgGAM groups vs the control and sham groups, and ** denotes significant differences between the CLP-IgG group vs the CLP and CLP-IgGAM groups. *Vertical bars* indicate standard deviations

Real-time PCR analysis of brain samples showed comparable C1qa, C9, complement inhibitor CD55, and CD59 expression levels at days 1 and 10. As an exception, cerebral C3 expression levels showed trends towards transient decline on the first day in the IgG and IgGAM treatment groups (Fig. 1). Regardless of treatment status, C3a receptor expression levels were significantly increased in all CLP groups on day 1 and showed a sharp decline on day 10. C5a receptor expression levels showed a marked increase in all CLP groups on day 1. On day 10, C5a receptor levels remained high in the CLP group with no treatment and with IgGAM treatment as compared to those in the control and sham groups. By contrast, C5a receptor expression levels of CLP rats with IgG treatment were significantly lower than those of the other two CLP groups and comparable to those of the control and sham groups (Fig. 1).

**Fig. 2:**
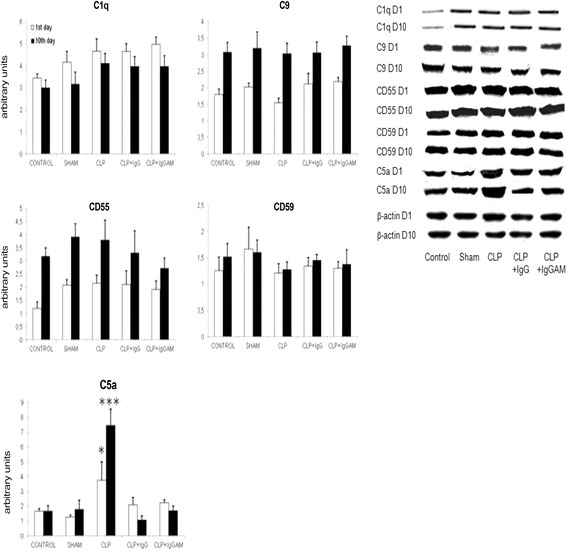
Brain expression levels of complement factors and complement inhibitors evaluated by immunoblotting, quantified by measuring band intensity with ImageJ, normalized by β-actin expression, and expressed as arbitrary units.*p < 0.05; ***p < 0.001. In the C5a panel, * and *** denote significant differences between the CLP group vs the other groups at the 1st (*white*) and 10th (*black*) days of the experiment, respectively. *Vertical bars* indicate standard deviations. *Lower panel* shows representative immunoblotting bands for each group and time point

In immunoblotting analysis performed to confirm real-time PCR results, in close resemblance to mRNA expression levels, no significant difference could be found among treatment arms by means of brain C1q, C9, CD55, and CD59 expression levels on days 1 and 10. By contrast, C5a levels were significantly elevated in rats with CLP on day 1. While the cerebral C5a levels of the CLP rats continued increasing on day 10, those of the IgG- and IgGAM-treated rats kept remaining at low levels that were comparable to those of the control and sham groups (Fig. 2).

### Reduced apoptotic factor expression in IVIgG- and IgGAM-administered rats

**Fig. 3:**
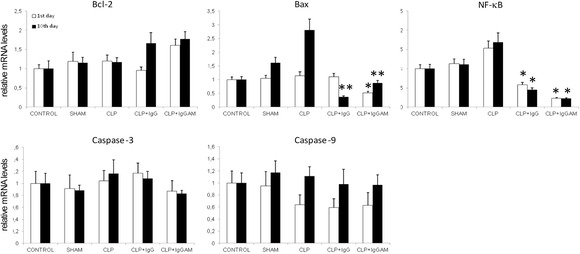
Brain mRNA expression levels of various apoptosis-related factors evaluated by real-time PCR and expressed as fold changes relative to the control group. *p < 0.05; **p < 0.01; the *asterisks* denote significant differences between the CLP-IgG and CLP-IgGAM groups vs the other groups. *Vertical bars* indicate standard deviations

**Fig. 4:**
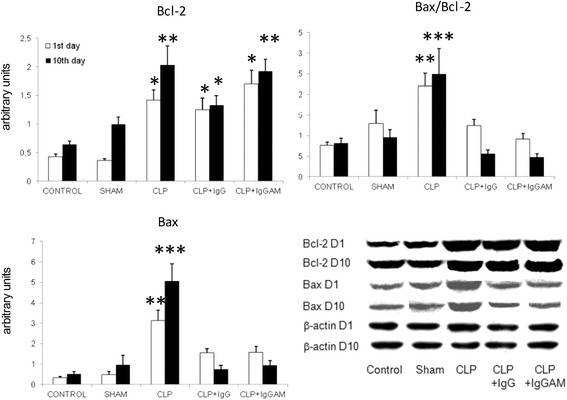
Brain expression levels of Bax, Bcl-2, and Bax/Bcl-2 ratios evaluated by immunoblotting, quantified by measuring band intensity with ImageJ, normalized by β-actin expression, and expressed as arbitrary units. *p < 0.05; **p < 0.01; ***p < 0.001. In the Bcl-2 panel, the *asterisks* denote significant differences between CLP, CLP-IgG, and CLP-IgGAM groups vs the control and sham groups; in the Bax and Bax/Bcl-2 panels, the *asterisks* denote significant differences between the CLP group vs the other groups. *Vertical bars* indicate standard deviations. *Lower panel* shows representative immunoblotting bands for each group and time point

mRNA expression levels of apoptotic molecules Bax and NF-κB were significantly lower in the brain samples of IgG- and IgGAM-administered rats as compared to those of the control, sham, and CLP groups. Anti-apoptotic Bcl-2 expression levels showed marked elevation especially on the 10th day. However, these differences did not attain statistical significance. There were no differences among study groups by means of expression levels of caspase 3 and caspase 9 (Fig. 3). Similarly, in western blot studies, Bcl-2 expression levels were significantly elevated in all CLP groups with or without treatment as compared to the control and sham groups. There were no significant differences between expression levels of Bcl-2 among CLP groups. Bax expression levels and Bax/Bcl-2 ratios were significantly elevated in CLP rats without treatment as compared to all other groups. Notably, in IgG- and IgGAM-administered rats, expression levels of Bax and Bax/Bcl-2 ratios were comparable to those of the control and sham groups (Fig. 4).

### IgG and IgGAM administration reduces apoptosis and glial proliferation

**Fig. 5:**
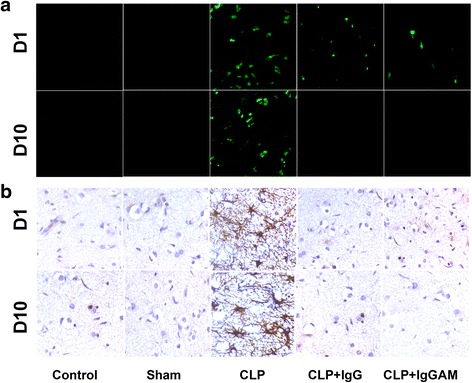
Representative immunofluorescence (**a**) and immunohistochemistry (**b**) images demonstrating the apoptosis (evaluated by TUNEL staining, **a**) and gliosis (evaluated by glial fibrillary acidic protein antibody staining, **b**) status of different treatment arms at day 1 (D1) and day 10 (D10) of the experiment. Each section roughly corresponds to the same cerebral cortex region (original magnification ×100)

Serial sections were obtained from one hemisphere of each rat, and the entire hemisphere was screened for apoptotic cells, infiltrating immune cells, and reactive gliosis. The CLP rats with no IVIg treatment showed markedly increased apoptotic cell numbers and intense reactive gliosis in all regions of the central nervous system. By contrast, no or very few apoptotic cells and reactive glial cells were observed in the brain samples of the control, sham, and CLP rats treated with IgG or IgGAM (Fig. 5a, b). H&E staining did not show any infiltrating cells in the brain samples. No immune reactivity was observed in immunohistochemistry studies performed to show potential infiltrating CD3, CD4 or CD8+ T cells, CD19+ B cells, and CD11b/c+ neutrophils/macrophages (Additional file 2: Table S2), while the control rat spleen tissue sections showed abundant reactivity with these markers (not shown).

## Discussion

The main finding of this study is that IVIg treatment reduces apoptotic cell death and glial cell proliferation and suppresses C5a activity in the central nervous system. Our results suggest that both IgG and IgGAM exert this protective effect by reducing C5a activity and proapoptotic NF-κB and Bax expressions thereby inhibiting major inflammation and apoptosis cascades.

Numerous clinical trials have examined the efficacy of IVIg in various autoimmune and degenerative diseases such as chronic inflammatory demyelinating polyneuropathy, multifocal motor neuropathy, Guillain–Barré syndrome, and Alzheimer’s disease [9, 10]. Effects of IVIg treatment on sepsis-induced polyneuropathy were previously reported in a retrospective trial [11]. We recently demonstrated that IVIg treatment protects the BBB integrity, inhibits CLP-induced sickness behavior, and improves survival in septic animals [6, 12].

There is increasing evidence that innate immunity disturbance in sepsis-induced organ dysfunction might be linked to the uncontrolled activation of the complement system [13, 14]. Also, C5a and C5a receptor expression levels were reported to be elevated in the lung, liver, kidney, pituitary gland, and heart during sepsis [5, 15]. In our study, significantly elevated levels of C5a were also found in the brain samples of the rats with sepsis. Likewise, C5a receptor expression levels showed a marked increase in all CLP groups on day 1 and remained high in the CLP group with no treatment. Although systemic complement activity was reduced in the IVIg-treated rats, cerebral complement and complement inhibitor factor levels were mostly comparable among treated and non-treated groups. As an exception, the IVIg-treated rats showed a striking downregulation of C5a and C5a receptor on day 10. Our results suggest that similar to other sepsis-afflicted tissues, C5a plays a major role in sepsis-induced destruction of the nervous system and apparently IVIg treatment reduces complement-based anaphylatoxin activity and ameliorates septic encephalopathy presumably through this mechanism of action.

BBB breakdown, abnormal neurotransmitter, amino acid derangements, and apoptosis have been documented to occur during sepsis and affect brain function [16–20]. Apoptosis is particularly a prominent feature in patients and animals with sepsis. Sepsis-associated apoptosis has been linked to C5a–C5a receptor interaction, which leads to organ dysfunction, immunosuppression, and lethality [16–19]. Apoptosis-related proteins have been shown in the cerebellum, hippocampus, astrocytes, and ependymal cells [21, 22]. In line with previous studies, the brain samples of the septic rats showed concomitantly increased C5a, C5a receptor expression, and apoptosis. While our results demonstrated no change in the caspase-dependent apoptosis pathway factors, Bax/Bcl-2 ratio, a widely used index of apoptosis, was significantly elevated in septic rats with no treatment, suggesting that sepsis-induced activation of neuronal apoptosis might occur through non-caspase-dependent pathways.

Experimental studies of the sepsis model with LPS challenge the demonstrated release of proinflammatory mediators produced by microglial activation [23, 24]. It was also reported that microglial activation is triggered by the activation of TLRs or by signals from apoptotic cells [25]. It has been suggested that glial activation not only might be the potential cause of acute, reversible alterations in the mental status, such as delirium, but also may lead to long-term cognitive dysfunction [26]. In our CLP model, the brain samples of the septic rats showed markedly increased gliosis. By contrast, cerebral apoptosis and gliosis were significantly diminished in parallel in the septic rats treated with IgG and IgGAM. The link between C5a receptor activation and development of apoptosis and gliosis is well known. C5a−C5a receptor interaction is known to trigger NF-κB activation [27, 28], and increased expression levels of C5a and NF-κB have been associated with enhanced apoptosis and gliosis [29–33]. Moreover, toxic products released by active glial cells might cause neuronal apoptosis [34]. Parallel and simultaneous elevation of apoptosis, gliosis, C5a, C5a receptor, and NF-κB expression levels in the brain tissues of septic rats and exactly similar trend of decrease in the levels of the same factors following IVIg treatment suggest that septic encephalopathy is caused by anaphylatoxin-mediated activation of inflammation pathways and glial cells, which ultimately induce neuronal apoptosis. IVIg treatment appears to ameliorate septic encephalopathy by interrupting this chain of action.

As in our previous experimental trials, we used two different types of immunoglobulin preparations (IgG and IgGAM) that are available in clinical practice. Both in vivo and in vitro trials in sepsis immunology demonstrated a trend towards a better activity of IgM in comparison to IgG [6]. Among immunoglobulin types, pure IgM was also shown to be most effective in preventing complement disposition, followed by IgG [35]. IgGAM preparation showed a better effect on the BBB integrity than a standard IgG preparation in CLP-induced septic model rats [6]. However, both immunoglobulin preparations were equally effective in reversing behavioral deficits induced by sepsis [12]. Similarly, in the current study, there were no remarkable differences between the clinical and molecular effects of two preparations with the exception that IVIgG treatment exerted a more pronounced and prolonged suppression effect on cerebral C5a receptor expression than IVIgGAM treatment. Thus, the influence of excess IgM in the IgGAM preparation on C5a receptor expression needs to be further investigated.

As usual, animal models might not strictly mimic the human disorder and thus the validity of our results need to be confirmed using postmortem brain tissue and cerebrospinal fluid samples of septic encephalopathy patients. Another limitation of our study is the absence of mechanistic studies that would more firmly demonstrate the crucial involvement of C5aR-activated intracellular pathways in septic encephalopathy. Future animal model experiments performed with specific C5aR and NF-κB agonists/antagonists or C5aR-deficient mice might more robustly disclose the significance of these pathways. C5a, C5aR, and NF-κB, which were shown to be the key molecules in septic encephalopathy pathogenesis, might also be utilized as potential targets for future treatment trials of septic encephalopathy. Numerous C5aR antagonists are currently available and have been tested for treatment of inflammatory disorders [36]. The efficacy of these molecules needs to be scrutinized using the septic encephalopathy animal model in future studies.

## Conclusions

The results of the present study indicate that IVIg treatment exerts its beneficial effects on sepsis-induced neuronal dysfunction primarily through reduction of C5a-mediated gliosis and apoptosis. Thus, our results also suggest that novel treatment methods based on interruption of C5a−C5a receptor interaction might ameliorate septic encephalopathy and presumably chronic cognitive dysfunction observed in sepsis survivors. The exact apoptosis, survival, and inflammation pathways involved in sepsis-induced neuronal dysfunction still remain to be elucidated.

## Additional files

Additional file 1: Table S1. Quantitative polymerase chain reaction primers. (DOCX 13.9 KB)
Additional file 2: Table S2. Primary antibodies used in western blot (WB) and immunohistochemistry (IHC) studies. (DOCX 11.16 kb)
Additional file 3: Figure S1. Serum C3, soluble C5b-9 levels, and total complement activity (CH50) at 1st (white) and 10th (black) days of the experiment. *, *p* < 0.05; **, *p* < 0.01; asterisks denote significant differences between the CLP + IgG or CLP + IgGAM groups vs the other groups. Vertical bars indicate standard deviations. (TIF 398 kb)
