# Supplementary material for: Neuroprotective effects of intravenous immunoglobulin are mediated through inhibition of complement activation and apoptosis in a rat model of sepsis
Source: Intensive Care Med Exp. 2017 Jan 5;5:1. doi: 10.1186/s40635-016-0114-1 (PMC5215999; doi:10.1186/s40635-016-0114-1)
Supplement: Additional file 1: Table S1. — Quantitative polymerase chain reaction primers. (DOCX 13.9 KB) [file 40635_2016_114_MOESM1_ESM.docx]

**Additional file 1: Table S1.** Quantitative polymerase chain reaction primers.

| **Primer name** | **Sequence 5′-3′** |
| --- | --- |
| CD55 F | GGCACCGTCTCTTCTACCTG |
| CD55 R | AGTTTGGCGTGGCATTAGGA |
| CD59 F | CTTTGTCACTTCGGAGGGCT |
| CD59 R | TCCTCCTGGTCCACTCACTT |
| Bcl-2 F | GGTGAACTGGGGGAGGATTG |
| Bcl-2 R | AGAGCGATGTTGTCCACCAG |
| Caspase-3 F | TACTCTACCGCACCCGGTTA |
| Caspase-3 R | CGTACAGTTTCAGCATGGCG |
| Caspase-9 F | AGACCATGGCTTTGAGGTGG |
| Caspase-9 R | CTTGGGCCTTCCTGGTATGG |
| Bax F | GCTGACATGTTTGCAGACGG |
| Bax R | GTGTCCAGCCCATGATGGTT |
| NfKb1 F | AAAAACGCATCCCAAGGTGC |
| NfKb1 R | AAGCTCAAGCCACCATACCC |
| C1qA F | TACCAGGGTACTGAAGCCGA |
| C1qA R | TAAAACCCCAGCCCCTTCAC |
| C3 F | GCGGTACTACCAGACCATCG |
| C3 R | CTTCTGGCACGACCTTCAGT |
| C9 F | CGCAAACCTTGGAACGTAGC |
| C9 R | AAACTCGTGGTCCTGTCTCG |
| C3a receptor F | GCCCCATCCCAGATGTTTGA |
| C3a receptor R | CACATGGCCGATTCAGGGAT |
| C5a receptor F | ATGGGAGACTCCGAAGGTCA |
| C5a receptor R | AGGTATCCATCGTGGACCGA |
| Beta-actin F* | CCGCGAGTACAACCTTCTTG |
| Beta-actin R* | CAGTTGGTGACAATGCCGTG |
| HPRT1 F* | GTCAAGCAGTACAGCCCCAA |
| HPRT1 R* | TGGCCACATCAACAGGACTC |
| UBC F* | ACACCAAGAAGGTCAAACAGGA |
| UBC R* | CACCTCCCCATCAAACCCAA |

HPRT, hypoxanthine-guanine phosphoribosyltransferase; UBC, ubiquitin C; NF-κB, nuclear factor kappa-light-chain-enhancer of activated B cells; Bcl-2, B-cell lymphoma 2; Bax, Bcl-2-associated X protein; F, forward; R, reverse.

* Indicates housekeeping genes.
