# Supplementary material for: Neuroprotective effects of intravenous immunoglobulin are mediated through inhibition of complement activation and apoptosis in a rat model of sepsis
Source: Intensive Care Med Exp. 2017 Jan 5;5:1. doi: 10.1186/s40635-016-0114-1 (PMC5215999; doi:10.1186/s40635-016-0114-1)
Supplement: Additional file 2: Table S2. — Primary antibodies used in western blot (WB) and immunohistochemistry (IHC) studies. (DOCX 11.16 kb) [file 40635_2016_114_MOESM2_ESM.docx]

**Additional file 2: Table S2.**Primary antibodies used in western blot (WB) and immunohistochemistry (IHC) studies.

| **Antibody/species** | **Application** | **Dilution** | **Source** |
| --- | --- | --- | --- |
| CD3/rabbit | IHC | 1:100 | Abcam, Cambridge, MA, USA |
| CD4/mouse | IHC | 1:100 | Abcam, Cambridge, MA, USA |
| CD8/mouse | IHC | 1:200 | Abcam, Cambridge, MA, USA |
| CD19/mouse | IHC | 1:100 | Abcam, Cambridge, MA, USA |
| CD11b/c/mouse | IHC | 1:200 | Abcam, Cambridge, MA, USA |
| GFAP/rabbit | IHC | 1:500 | Abcam, Cambridge, MA, USA |
| C1q/rabbit (26 kDa) | WB | 1:100 | Biorbyt, Cambridge, UK |
| C9/rabbit (42 kDa) | WB | 1:1000 | Biorbyt, Cambridge, UK |
| C5a/rabbit (120 kDa) | WB | 1:1000 | BosterBio, Pleasanton, CA, USA |
| CD55/rabbit (70 kDa) | WB | 1:1000 | Santa Cruz, Dallas, TX, USA |
| CD59/mouse (20 kDa) | WB | 1:1000 | Santa Cruz, Dallas, TX, USA |
| Bcl-2/rabbit (26 kDa) | WB | 1:1000 | Santa Cruz, Dallas, TX, USA |
| Bax/rabbit (23 kDa) | WB | 1:1000 | Santa Cruz, Dallas, TX, USA |
| β-actin/rabbit (42 kDa) | WB | 1:1000 | Biorbyt, Cambridge, UK |

kDa values in brackets indicate band sizes observed during western blot experiments.
